# Differential regulation of follicle stimulating hormone by activin A and TGFB1 in murine gonadotropes

**DOI:** 10.1186/1477-7827-3-73

**Published:** 2005-12-29

**Authors:** A Jesse Gore, Daniel P Philips, William L Miller, Daniel J Bernard

**Affiliations:** 1 Department of Molecular and Structural Biochemistry, Box 7622, North Carolina State University, Raleigh, NC 27695-7622, USA; 2 Center for Biomedical Research, Population Council, 1230 York Ave., New York, NY 10021, USA; 3 The Rockefeller University, 1230 York Ave., New York, NY 10021, USA

## Abstract

**Background:**

Activins stimulate the synthesis of follicle stimulating hormone (FSH) in pituitary gonadotropes, at least in part, by inducing transcription of its beta subunit (*Fshb*). Evidence from several laboratories studying transformed murine LbetaT2 gonadotropes indicates that activins signal through Smad-dependent and/or Smad-independent pathways, similar to those used by transforming growth factor beta-1 (TGFB1) in other cell types. Therefore, given common intracellular signaling mechanisms of these two ligands, we examined whether TGFBs can also induce transcription of *Fshb *in LbetaT2 cells as well as in purified primary murine gonadotropes.

**Methods:**

Murine *Fshb *promoter-reporter (-1990/+1 m*Fshb*-luc) activity was measured in LbetaT2 cells treated with activin A or TGFB1, and in cells transfected with either activin or TGFB receptors. The ability of the ligands to stimulate phosphorylation of Smads 2 and 3 in LbetaT2 cells was measured by western blot analysis, and expression of TGFB type I and II receptors was assessed by reverse transcriptase polymerase chain reaction in both LbetaT2 cells and primary gonadotropes purified from male mice of different ages. Finally, regulation of endogenous murine *Fshb *mRNA levels by activin A and TGFB1 in purified gonadotropes and whole pituitary cultures was measured using quantitative RT-PCR.

**Results:**

Activin A dose-dependently stimulated -1990/+1 m*Fshb*-luc activity in LbetaT2 cells, but TGFB1 had no effect at doses up to 5 nM. Similarly, activin A, but not TGFB1, stimulated Smad 2 and 3 phosphorylation in these cells. Constitutively active forms of the activin (Acvr1b-T206D) and TGFB (TGFBR1-T204D) type I receptors strongly stimulated -1990/+1 m*Fshb*-luc activity, showing that mechanisms down stream of Tgfbr1 seem to be intact in LbetaT2 cells. RT-PCR analysis of LbetaT2 cells and whole adult murine pituitaries indicated that both expressed *Tgfbr1 *mRNA, but that *Tgfbr2 *was not detected in LbetaT2 cells. When cells were transfected with a human TGFBR2 expression construct, TGFB1 acquired the ability to significantly stimulate -1990/+1 m*Fshb*-luc activity. In contrast to LbetaT2 cells, primary murine gonadotropes from young mice (8–10 weeks) contained low, but detectable levels of *Tgfbr2 *mRNA and these levels increased in older mice (1 yr). A second surprise was the finding that treatment of purified primary gonadotropes with TGFB1 decreased murine *Fshb *mRNA expression by 95% whereas activin A stimulated expression by 31-fold.

**Conclusion:**

These data indicate that TGFB1-insensitivity in LbetaT2 cells results from a deficiency in *Tgfbr2 *expression. In primary gonadotropes, however, expression of *Tgfbr2 *does occur, and its presence permits TGFB1 to inhibit *Fshb *transcription, whereas activin A stimulates it. These divergent actions of activin A and TGFB1 were unexpected and show that the two ligands may act through distinct pathways to cause opposing biological effects in primary murine gonadotropes.

## Background

Follicle-stimulating hormone (FSH) synthesis, secretion, and action are critical for reproductive function in mammals, particularly in females [1-3]. FSH production is regulated by a variety of neuroendocrine, intra-pituitary, and gonadal factors. Arguably, the most potent and selective stimulators of FSH synthesis are the activins, members of the transforming growth factor beta (TGFB) superfamily. Within the anterior pituitary, activins (activin B, in particular) act in paracrine/autocrine fashion to induce expression of the FSH beta (*Fshb*) subunit [4-9], the rate-limiting step in mature FSH production.

Several other factors that regulate FSH synthesis appear to have their actions via synergy with or perturbation of endogenous activin signaling. For example, activins synergistically stimulate rat and sheep *Fshb *transcription with gonadotropin releasing hormone (GNRH1) via cross-talk between activin and GNRH1 signaling pathways as well as through regulation of GNRH1 receptor expression [10-13]. Testicular androgens regulate *Fshb *transcription both directly and indirectly, although these effects vary across species [14]. In sheep, the direct actions of androgens on transcription appear to require intact activin signal transduction mechanisms [15]. Follistatins (FST) inhibit FSH production by binding activins and blocking the latter from interacting with their cell surface receptors [16,17]. Similarly, gonadal inhibins suppress FSH synthesis via antagonism of activins; in this case through competition with activins for binding to activin type II receptors [18-22]. Thus, many of the endocrine and paracrine factors known to affect FSH production do so through an interaction with or disruption of activin signaling. These and other data [23-25] indicate that the activins are critical for normal FSH regulation.

Both activins and TGFBs bind hetero-tetrameric receptor complexes consisting of ligand specific type I and type II receptor serine/threonine kinases [26,27]. Activins bind one of two type II receptors, ACVR2A or ACVR2B, which then recruit and phosphorylate the activin type IB receptor, ACVR1B or ALK4. In analogous fashion, TGFB1 binds TGFBR2, which recruits and phosphorylates TGFBR1 (also known as ALK5). Once activated, ACVR1B and TGFBR1 can phosphorylate Smad2 and Smad3 on C-terminal serine residues [28,29], and can also activate TGFB-activated kinase 1 (TAK1) [30,31]

In rodents, activins stimulate *Fshb *subunit gene transcription through both immediate-early and indirect (delayed or late) signaling pathways [30,32-34]. There is evidence to implicate Smads in *Fshb *gene transcription because they are rapidly phosphorylated and trans-located to the nucleus rapidly following activin A treatment [32,35-37]. In rats and mice, interference with Smad2 or Smad3 signaling impairs activin A-regulated *Fshb *transcription [13,32,33,35,37]. However, these proteins seem to play less important roles in activin A-induced *Fshb *transcription in sheep and humans [30,33], where Smad-independent mechanisms mediated by TGFB-activated kinase 1 (TAK1) appear to be critical for the former.

Like activins, the TGFB isoforms 1, 2, and 3 also phosphorylate and activate Smad2, Smad3 and TAK1 [28,29,31]. TGFB1 is produced within rat pituitary lactotropes [38,39]. Therefore, it is possible that TGFBs, acting in a paracrine manner, may also stimulate rodent *Fshb *transcription in gonadotropes via a similar Smad2/3- and/or TAK1-dependent mechanism. If this occurs, however, how could gonadotrope cells discriminate intracellularly between activin and TGFB-generated signals, specifically with respect to FSH regulation? This is an important question, in light of the fact that various physiological mechanisms that have evolved to spatially and temporally restrict activin's actions do not affect TGFB signaling [16,40]. For example, ovarian inhibin B production and action during metestrus and diestrus are critical for the suppression of activin-stimulated FSH production at these times of the rat estrous cycle [41,42]. Inhibin B may play a similar role during the follicular phase of the human menstrual cycle [43,44]. In addition, FST is dynamically regulated in the pituitary across the rat estrous cycle and its patterns of expression appear to be critical for the proper timing of the secondary FSH surge on the morning of proestrus [45,46]. Neither the inhibins nor FST suppress TGFB1 actions [19,47-49]. Therefore, antagonism of activins' stimulation of FSH by these proteins could theoretically be circumvented by unfettered TGFB1 stimulation of Smad2/3- and/or TAK1-dependent signaling mechanisms. We, therefore, examined TGFB1-regulated expression of murine *Fshb *subunit transcription to determine whether or not gonadotropes have evolved a mechanism to discriminate between the activin and TGFB ligands.

## Methods

### Reagents and constructs

Human recombinant (rh-) TGFB1, rh-activin A, recombinant mouse (rm)-follistatin 288 were purchased from R&D systems (Minneapolis, MN). Dulbecco's modified Eagle medium (DMEM), Lipofectamine/Plus, Lipofectamine 2000, gentamycin, and Trizol were from Invitrogen (Carlsbad, CA). Fetal bovine serum (FBS) was from JRH Biosciences (Lenexa, KS). The anti-Smad3 affinity purified rabbit polyclonal antibody was purchased from Zymed (South San Francisco, CA). Anti-Smad2/3 and phospho-Smad2 affinity purified rabbit polyclonal antibodies were purchased from Upstate Biotech (Waltham, MA). The phospho-Smad3 rabbit polyclonal antibody was a generous gift of Dr. Michael Reiss (Robert Wood Johnson Medical School). Protease inhibitor tablets (CompleteMini) were purchased from Roche (Indianapolis, IN). Deoxynucleotide triphosphates (dNTPs), MMLV reverse transcriptase, random primer hexamers, and Taq polymerase were from Promega (Madison, WI). The -1990/+1 m*Fshb*-luc reporter and constitutively active HA-rat ALK4 (Acvr1b) were described previously [32]. HA-human TGFBR1(T204D) was provided by Dr. Peter Scheiffele (Columbia University). The 3TP-luc reporter and HA-human TGFBR2 expression construct were gifts of Dr. Joan Massague (Memorial Sloan Kettering Cancer Center).

### Primary gonadotropes

Pituitaries from mice containing the ovine *FSHB-H2K*^*K *^transgene were used for gonadotrope purification. Young mice (8–10 weeks old; 10–18 mice) or older mice (1 year old; 12 mice) were killed and their pituitaries were dispersed and the gonadotropes were purified as reported [50]. Cells that did not attach to the magnetic column were labeled "gonadotrope-depleted," while cells eluted from the column after removal of the magnetic field were labeled "gonadotropes." Cell counts were obtained for all cell types using a hemocytometer. Equal numbers of cells were cultured in medium 199 (Gibco) with 10% charcoal-treated sheep serum and antibiotics/antimycotics as reported [51]. Gonadotropes and gonadotrope-depleted cells purified from younger mice were plated in triplicate at a density of 18,000 cells per well (first experiment) or 30,000 cells per well (second and third experiments). Cells isolated from older mice were plated in triplicate at a density of 50,000 cells per well in two separate experiments. For treatments with activin A or TGFB1, purified gonadotropes or whole pituitary cells were plated in triplicate at a density of 10,000 cells per well in three separate experiments. Gonadotropes, gonadotrope-depleted cells, and whole pituitary cells were cultured in 200 μl of media in 96 well Primaria culture plates (Becton Dickinson & Co, Franklin Lakes, NJ). Cells were incubated at 37° under 5% CO_2 _for 48 hrs prior to RNA isolation. All mice were handled in accordance with the rules and regulations of the Institutional Animal Care and Use Committee of North Carolina State University.

### Cell culture and transfection

Immortalized murine gonadotrope LβT2 cells were provided by Dr. Pamela Mellon (University of California, San Diego) and were cultured as described previously [32]. Murine fibroblast NIH3T3 cells were obtained from Dr. Patricia Morris (Population Council) and were cultured in DMEM/10% FBS. Cells were plated in 6- or 24-well plates at densities of 1 × 10^6 ^or 2 × 10^5 ^cells per well, respectively, approximately 36 hr prior to transfection. Cells were transfected with Lipofectamine/Plus or Lipofectamine 2000 following the manufacturer's instructions. Reporter plasmids were transfected at 1 μg (6-well) or 450 ng (24-well) per well. Expression plasmids were introduced at 300 ng (24-well) per well. In all experiments, the total amount of DNA added was balanced across treatments with empty expression vector pcDNA3.0 (Invitrogen).

In reporter experiments including ligand treatment, activin A or TGFB1 were added at the indicated concentrations for approximately 24 hr. Cells were washed with 1× PBS and lysed in 1× Passive Lysis Buffer (Promega). Luciferase assays were performed on a Luminoskan Ascent luminometer (Thermo Labsystems, Franklin, MA) as described [32]. All transfection conditions were performed in triplicate and each experiment performed 2–3 times.

### Western blotting

LβT2 and NIH3T3 cells were seeded at 7 or 4 × 10^5 ^cells per well, respectively, in 6-well plates. After 24–48 hr., cells were washed with serum-free DMEM and then incubated in the same medium overnight. The following day, cells were treated with the indicated concentrations of activin A or TGFB1 in fresh serum-free DMEM for 1 hr. After a wash with PBS, whole cell lysates prepared in RIPA buffer containing protease inhibitors. Equivalent amounts of protein were separated by 8% Tris-glycine SDS-PAGE and transferred to Protran (Schleicher & Schuell, Keene, NH). Filters were probed with anti-phospho-Smad2, anti-phospho-Smad3, anti-Smad2/3, or anti-Smad3 using previously described methods [32].

### Semi-quantitative RT-PCR

Total RNA was extracted from adult female CD-1 murine pituitaries and LβT2 cells using Trizol following the manufacturer's instructions. Four μg of total RNA were reverse transcribed (RT) into cDNA using 100 ng random hexamer primers and 100 U MMLV-RT. A second set of samples was processed similarly, except the RT enzyme was omitted (no RT) as a control for contaminating genomic DNA in the RNA samples. One-tenth of each RT or RT- reaction was used as template in PCRs for *Tgfbr1 *(503 bp) and *Tgfbr2 *(536 bp). PCR was run using the following conditions for 35 cycles: 94C for 30 sec, 53C for 30 sec, and 72C for 30 sec. Reactions contained 0.4 pmol of each primer, 200 μM dNTPs, 1.5 mM MgCl_2_, 1× PCR buffer, and 2.5 U Taq polymerase. Following a final 7 min. extension step at 72C, one-fifth of each reaction was resolved on a 1 % agarose gel containing ethidium bromide. Gels were photo-documented using a digital camera interfaced with an IBM ThinkPad computer running the Kodak Digital Science 1D software (v.2.0.2) software. Reactions with no template (H_2_O only) were used to confirm the absence of contaminating DNA in the reagents. The primer sets for *Tgfbr1 *and *Tgfbr2 *were as follows: *Tgfbr1*, (forward) AACCTGTTGTATTGCAGACTT and (reverse) GAGCAGAGTTCCCACGGTGT; *Tgfbr2*, (forward) TTGCCTGTGTGACTTCGGGCT and (reverse) CTATTTGGTAGTGTTCAGCGA.

### Real-Time RT-PCR (RT-rtPCR)

Total RNA from primary and LβT2 cells was isolated and converted to cDNA as reported [51]. Oligonucleotides for Taqman real-time PCR were designed for murine cDNA using software from Integrated DNA Technologies, Inc (Coralville, IA) for *Tgfbr1*, *Tgfbr2*, *Fshb *and prolactin (Table 1). Using the same oligonucleotides as described previously [50], murine *18s *ribosomal RNA served as the endogenous control. All Taqman probes were 5' -labeled with FAM and real-time PCR of all cDNA samples was performed at the same time. Real-time PCR was performed in duplicate on triplicate cDNA samples from both gonadotropes and gonadotrope-depleted cells using an iCycler (Bio-Rad, Inc). Samples were incubated at 95°C for 3 min, and then for 40 complete cycles (95°C for 30 sec, 55°C for 30 sec, and 72°C for 30 sec). There was a final extension step of 72°C for 3 min. Threshold cycle (C_T_) values were determined with Bio-Rad software and used for relative quantitation with the 2^-ΔΔCt ^method [52].

**Table 1:** Real Time RT-PCR primer and probe sequences

**Primer/Probe Set**		**Sequence**
*Tgfbr1*	Forward	5': CATTCACCACCGTGTGCCAAATGA
	Reverse	5': ACCTGATCCAGACCCTGATGTTGT
	Probe	5': AGATCGCCCTTTCATTTCAGAGGGCA
*Tgfbr2*	Forward	5': TCCCAAGTCGGATGTGGAAATGGA
	Reverse	5': TCGCTGGCCATGACATCACTGTTA
	Probe	5': AGCCCAGAAAGATGCATCCATCCACGTA
*Prolactin*	Forward	5': TCTCAAGGTCCTGAGGTGCCAAAT
	Reverse	5': CCATTGCACCCAAGCATGCACTGA
	Probe	5': ACAACTGCTAAACCCACATTCAGTCCA
*Fshb*	Forward	5': AGAGAAGGAAGAGTGCCGTTTCTG
	Reverse	5': ACATACTTTCTGGGTATTGGGCCG
	Probe	5': ATCAATACCACTTGGTGTGCGGGCTA
*18s *rRNA	Forward	5': GAAACTGCGAATGGCTCATTAA
	Reverse	5': GAATCACCACAGTTATCCAAGTAGGA
	Probe	5': ATGGTTCCTTTGGTCGCTCGCTCC

### Statistical analysis

The data from replicate luciferase assay experiments were highly similar and were pooled (n = 6 or 9 per treatment) for statistical analyses. Data are presented as fold-change from the control condition in each experiment. Differences between means were compared using one- or two-way analyses of variance followed by post-hoc Scheffe or Bonferroni tests (Systat 10.2, Richmond, CA). Comparisons of relative receptor mRNA expression in gonadotrope and gonadotrope-depleted cells in different age groups were performed with two-way ANOVAs of log-transformed data. *Fshb *mRNA levels were compared in one-way ANOVAs of log-transformed data. In all cases, significance was assessed relative to *p *< 0.05.

## Results

### Activin A but not TGFB1, stimulated *Fshb *transcription in LβT2 cells

**Figure 1:**
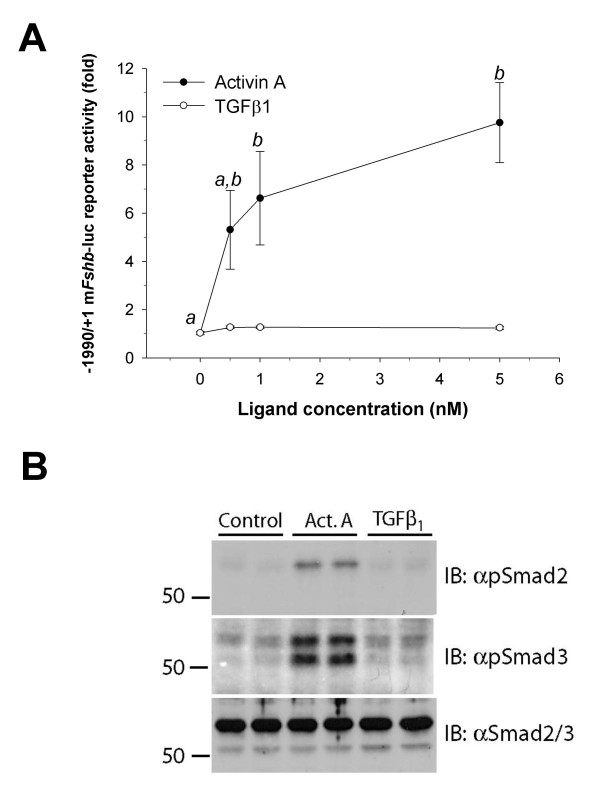
**TGFB1 fails to stimulate *Fshb *transcription or Smad2/3 phosphorylation in LβT2 cells. **A) LβT2 cells were seeded in 6-well plates and transfected with the murine -1990/+1 m*Fshb*-luc reporter. Following transfection, cells were treated with the indicated concentrations of activin A (closed circles) or TGFB1 for approximately 24 hours. Lysates were subjected to luciferase assays. Data points reflect mean (+/- SEM) fold-change in luciferase activity from the control condition (0 nM) in two experiments performed in triplicate (n = 6). Points with different letters differed significantly. B) LβT2 cells seeded in 6-well plates were treated with vehicle (control), 1.2 nM activin A or TGFB1 for 1 hour. Immunoblots (IB) of whole cell lysates were probed with rabbit anti-phospho-Smad2 (top), anti-phospho-Smad3 (middle), or anti-Smad2/3 (bottom) antibodies. Treatments were performed in duplicate. Numbers at the left are molecular weight standards in kDa.

**Figure 2:**
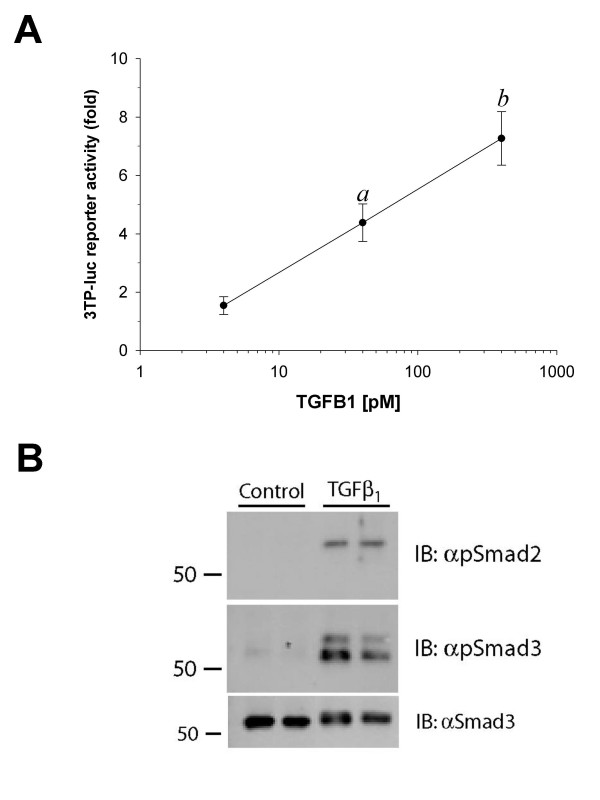
**TGFB1 stimulates 3TP-luc activity and Smad2/3 phosphorylation in NIH3T3 cells. **A) Murine fibroblast NIH3T3 cells were seeded in 24-well plates and transfected with the TGFB responsive promoter-reporter 3TP-luc. Following transfection, cells were treated with 4–400 pM TGFB1 for approximately 24 hours. Luciferase assays were performed as described. Data points reflect mean (+/- SEM) fold-change in luciferase activity from the control condition (0 pM, not pictured) in two experiments performed in triplicate (n = 6). The data are presented on a log-linear plot. Points with letters differed from control and points with different letters differed from one another. B) NIH3T3 cells seeded in 6-well plates were treated with vehicle (control) or 400 pM TGFB1 for 1 hour. Immunoblots on whole cell lysates were performed as described in the legend to Figure 1, except in the bottom blot an anti-Smad3 antibody was used in place of anti-Smad2/3. Treatments were performed in duplicate. Numbers at the left are molecular weight standards in kDa.

LβT2 cells were transfected with a murine -1990/+1 *Fshb *luciferase promoter-reporter construct (-1990/+1 m*FSHB*-luc) [32] and were treated with different concentrations of activin A or TGFB1 for approximately 24 hr. Whereas activin A dose-dependently stimulated reporter activity, TGFB1 had no effect at concentrations up to 5 nM (Fig. 1A). In addition, activin A, but not TGFB1, stimulated Smad 2 and 3 phosphorylation in these cells (Fig. 1B). In contrast, the same lot of TGFB1 at lower concentrations (4–400 pM) dose-dependently stimulated the activin/TGFB responsive promoter of 3TP-luc [53], and Smad2/3 phosphorylation in murine NIH3T3 fibroblast cells (Figs. 2A and 2B). Thus, the TGFB1 ligand was biologically active, but LβT2 cells were somehow insensitive to it.

### Constitutively active activin and TGFB type I receptors stimulated *Fshb *transcription in LβT2 cells

**Figure 3:**
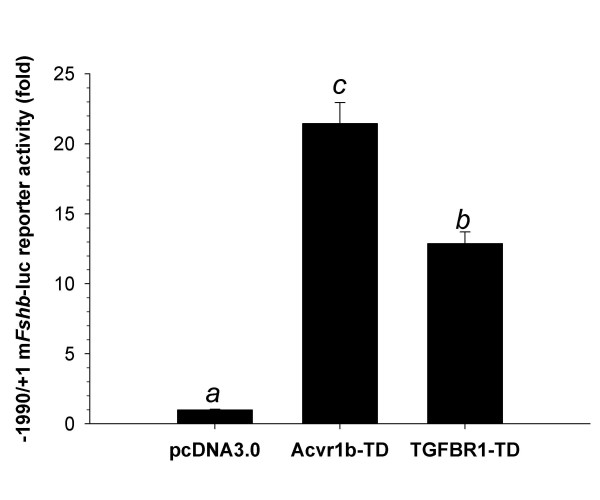
**Constitutively active activin and TGFB type I receptors stimulate *Fshb *transcription in LβT2 cells. **LβT2 cells seeded in 24-well plates were transfected with the -1990/+1 m*Fshb*-luc reporter and expression vectors for constitutively active forms of the rat activin (Acvr1b-TD) and human TGFB (TGFBR1-TD) type I receptors or with an empty expression vector (pcDNA3.0). Data reflect mean (+/- SEM) fold-change in luciferase activity from the control condition (pcDNA3.0) in two experiments performed in triplicate (n = 6). Bars with different letters differed significantly.

We previously showed that a constitutively active form of rat Acvr1b (T206D), which can stimulate Smad phosphorylation in the absence of activins and the type II receptors [54], stimulated murine *Fshb *promoter-reporter activity [32]. Here, we asked whether a constitutively active form of TGFBR1 (T204D; [55]) could similarly stimulate *Fshb *transcription in LβT2 cells. As shown in Figure 3, both rat Acvr1b-TD and human TGFBR1-TD potently stimulated -1990/+1m*Fshb*-luc. These data indicate that events downstream of TGFBR1 (whether Smad-dependent or Smad-independent; [30,56]) seem to be present in LβT2 cells and therefore that the cells' insensitivity to TGFB1 likely derives from a deficiency at the receptor level.

### LβT2 cells do not express the TGFB type II receptor, Tgfbr2

**Figure 4:**
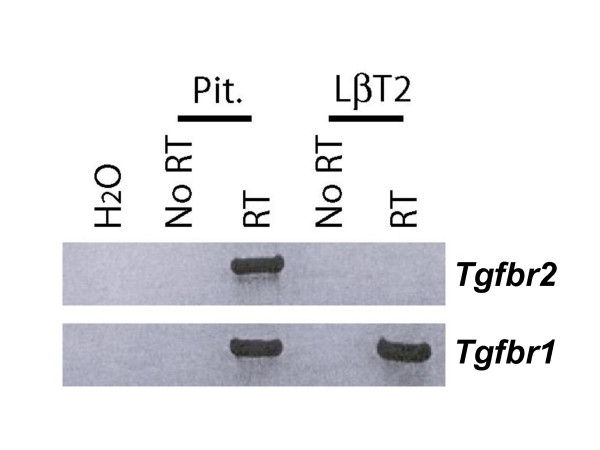
**LβT2 cells do not express *Tgfbr2 *mRNA. **RT-PCR analysis of TGFB receptor expression in adult female CD-1 murine pituitary gland and LβT2 cells. Whereas *Tgfbr1 *mRNA is expressed in both samples (bottom), *Tgfbr2 *is expressed in whole pituitaries but not in LβT2 cells (top). No amplicons were detected in negative control samples (i.e., H_2_O only or no RT).

We used RT-PCR to examine *Tgfbr2 *and *Tgfbr1 *mRNA levels in LβT2 cells compared to adult murine pituitary glands. Whereas both LβT2 cells and pituitary glands expressed *Tgfbr1 *mRNA, only the latter expressed *Tgfbr2 *(Fig. 4).

### Over-expression of TGFBR2 in LβT2 cells conferred TGFB1 responsiveness in LβT2 cells

**Figure 5:**
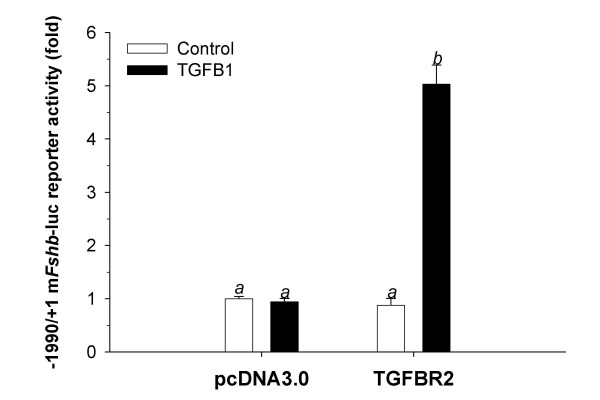
**TGFBR2 over-expression rescues TGFB responsiveness in LβT2 cells. **LβT2 cells seeded in 24-well plates were transfected with the -1990/+1 m*Fshb*-luc reporter and an expression vector for the human TGFBR2. Control wells were transfected with an empty expression vector, pcDNA3.0. Half of the cells in each condition were then treated with 400 pM TGFB1 for approximately 24 hr (filled bars). Data points reflect mean (+/- SEM) fold-change in luciferase activity from the control condition (pcDNA3.0, control) in three experiments performed in triplicate (n = 9). Bars with different letters differed significantly.

RT-PCR analysis indicated that LβT2 cells do not express *Tgfbr2 *mRNA. Therefore, these cells may not respond to TGFB1 because of a deficiency in this receptor. It is possible, however, that additional mechanisms contribute to TGFB1 insensitivity. To address this issue, we transfected LβT2 cells with a human TGFBR2 expression construct and examined TGFB1-stimulated *Fshb *transcription. Over-expression of the receptor alone had no effect on basal transcription, but made it possible for TGFB1 to stimulate -1990/+1 m*Fshb*-luc activity (Fig. 5). Therefore, a deficiency in *Tgfbr2 *expression appeared to account for the inability of LβT2 cells to respond to TGFB1.

### Primary murine gonadotropes exhibit low *Tgfbr2 *expression

LβT2 cells were derived from a pituitary tumor in a female transgenic mouse [57]. Whereas these cells show many of the features of fully differentiated gonadotropes, they are transformed cells and exhibit clear differences from gonadotropes *in vivo*. For example, basal *Fshb *expression is substantially lower in LβT2 cells than in gonadotropes (personal observations). Therefore, it is possible that the *Tgfbr2*-deficiency observed in LβT2 cells may not accurately reflect receptor expression in gonadotropes *in vivo*, though previous analyses in rats indicated that within the pituitary, *Tgfbr2 *expression is most abundant in lactotropes [58,59]. In order to examine receptor expression in murine gonadotropes, we purified this cell type from male mice, aged eight to ten weeks (young) or 1 year of age (old), using a recently described transgenic model [50]. Using real-time RT-PCR, prolactin (*Prl*) expression was examined to determine the level of purification of the gonadotropes from mixed primary pituitary cultures as described earlier [50]. The level of purity ranged from 97 % to 99% (data not shown).

**Figure 6:**
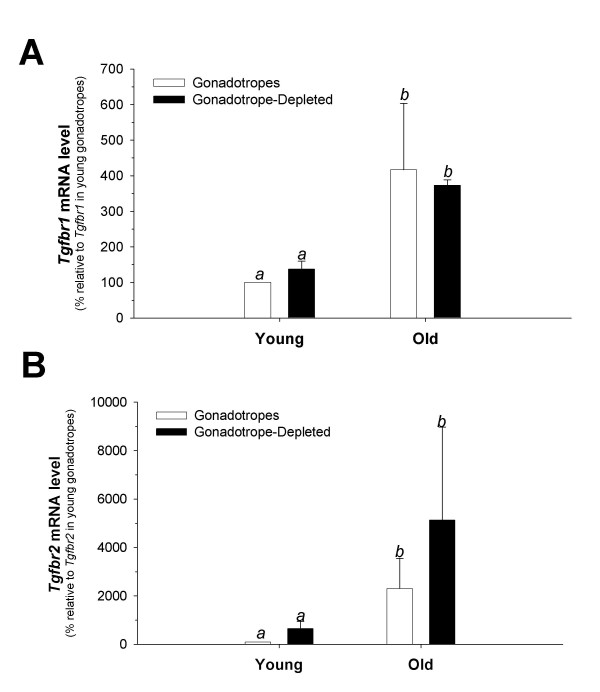
**Purified murine gonadotropes express *Tgfbr2 *at low levels. **Purified gonadotropes and gonadotrope-depleted pituitary cells were plated in triplicate in 96 well culture plates. After 48 hrs, total RNA was isolated and real-time RT-PCR was performed to examine *Tgfbr1 *and *Tgfbr2 *mRNA expression. Normalized threshold cycle (Ct) values were averaged and used to compare receptor expression in the different cell types and different age groups using the 2^-ΔΔCt ^method for quantitation. Data are presented as mean (+ SD) (A) *Tgfbr1 *or (B) *Tgfbr2 *mRNA levels relative to those in young murine gonadotropes (set to 100%). Data from young and old animals were from 3 or 2 independent experiments, respectively. Bars with different letters differed significantly. When averaged across age-groups, *Tgfbr2 *levels were higher in gonadotrope-depleted cells than in gonadotropes. Note the different scales of the *y*-axes in (A) and (B).

We then measured *Tgfbr1 *and *Tgfbr2 *mRNAs using real-time RT-PCR. *Tgfbr1 *mRNA was significantly higher in older than younger animals (*p *< 0.001), but did not differ significantly between gonadotropes and gonadotrope-depleted cells, nor was there a significant interaction between these two variables (Fig. 6A). In contrast to LβT2 cell data, *Tgfbr2 *mRNA was detected in gonadotropes but it was greater in older than younger animals (*p *< 0.001). *Tgfbr2 *mRNA was also higher in gonadotrope-depleted cells than pure gonadotropes across both age groups (*p *< 0.007) (Fig. 6B). In young mice, gonadotrope-depleted cells expressed *Tgfbr2 *6.5-fold higher than purified gonadotropes, whereas in the old mice the difference was reduced to 2.2-fold, but the interaction between cell type and age was not statistically significant. In the same assays, *Tgfbr2 *was undetectable in LβT2 cells, and *Tgfbr1 *in LβT2 cells was expressed at roughly 20% of *Tgfbr1 *in purified gonadotropes in young animals (data not shown).

### Activin A increased, and TGFB1 decreased, *Fshb *mRNA levels in murine gonadotropes and whole murine pituitary cultures

**Figure 7:**
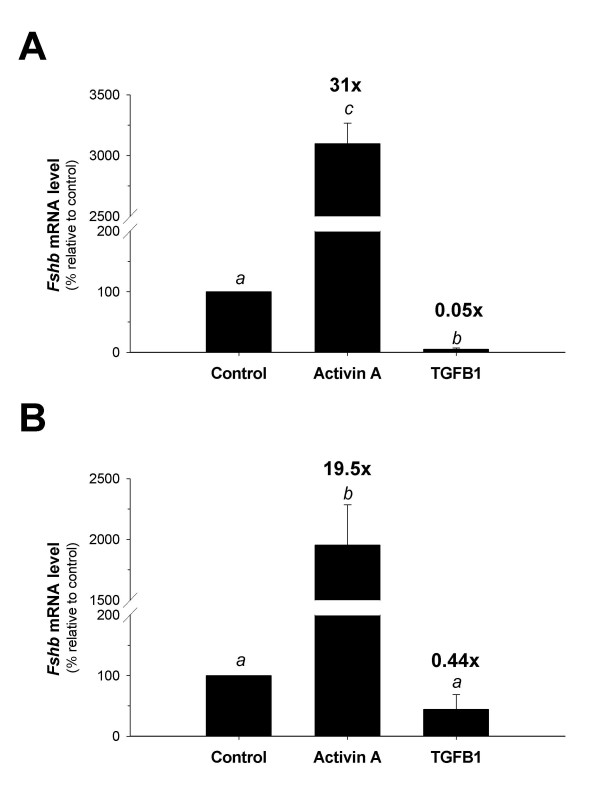
**Activin A stimulates, and TGFB1 inhibits, *Fshb *transcription in gonadotropes. **Purified gonadotropes (A) or whole pituitary cells (B) were plated in triplicate for control, activin A, or TGFB1 treatments in 96 well culture plates. After 24 hrs, cells were pre-treated with follistatin (250 ng/ml). After 24 hrs, all media was removed, and the cells were treated with control media, or media containing either activin A (60 ng/ml) or TGFB1 (60 ng/ml). After 24 hrs of treatment, total RNA was isolated and real-time RT-PCR was performed. Normalized threshold cycle (Ct) values were averaged and used to quantitate *Fshb *mRNA expression with the 2^-(ΔΔCt)^ method for quantitation. The mRNA levels were quantitated relative to the mRNA levels of *Fshb *in control cells for purified gonadotropes (A) or whole pituitary cells (B), which were normalized to 100 %. Plotted are the means (+/- SEM) for three experimental replicates. Bars with different letters differed significantly.

Because purified gonadotropes express *Tgfbr2*, we used real-time RT-PCR to examine the effects of activin A and TGFB1 on endogenous *Fshb *mRNA levels. Treatment of purified gonadotropes from younger mice with activin A resulted in a 31-fold stimulation of *Fshb *mRNA (Fig. 7A). Surprisingly, treatment with TGFB1 resulted in a significant 95 % reduction in *Fshb *mRNA levels (Fig. 7A). Similar results were obtained in gonadotropes isolated from older mice (data not shown). Importantly, treatment with TGFB1 did not appear to affect cell viability since there were no differences in the levels of *18s *rRNA between control and treated cells (data not shown), and no morphological changes of treated cells relative to control were observed (personal observations). In whole pituitary cultures, activin A and TGFB1 exerted similar effects on *Fshb *expression to those seen in purified gonadotropes, although their magnitudes were reduced (Fig. 7B). Activin A induction was 19.5-fold, and inhibition by TGFB1 was only 56% and was not statistically significant (*p *= 0.175, Scheffe post-hoc). Therefore, unlike LβT2 cells, primary murine gonadotropes are sensitive to TGFB1 and the ligand inhibits *Fshb *mRNA levels, perhaps by repressing transcription.

## Discussion

Activins regulate rodent and ovine *Fshb *transcription via Smad2/3- and/or TAK1-dependent intracellular signaling pathways [13,30,32,33,35,37,60]. Although TGFB isoforms also activate these pathways, we found that TGFB1 fails to regulate murine *Fshb *transcription in LβT2 cells, apparently because they do not express the TGFB type II receptor, Tgfbr2. However, when the cells were transiently transfected with the receptor, TGFB1 stimulated murine *Fshb *transcription. In striking contrast, we observed that gonadotrope cells purified from male mice expressed low levels of *Tgfbr2 *mRNA and that TGFB1 suppressed *Fshb *mRNA in these cells as well as in mixed murine pituitary cell cultures. Because over-expression of TGFBR2 allowed TGFB1 to stimulate the -1990/+1 m*Fshb*-luc construct in LβT2 cells, differences in TGFB1 responses observed between the cell line and purified gonadotropes do not appear to be attributable to differences in *Tgfbr2 *expression. Instead, the mechanisms through which TGFB1 inhibits *Fshb *expression in gonadotropes may be absent from LβT2 cells.

The identity of these inhibitory mechanisms is currently unknown, though opposing actions of activin A and TGFB1 have been noted in other cellular contexts [61,62]. Also, TGFB1 can inhibit its own prototypic signaling via TGFBR1 and Smad2/3, through an ACVRL1 (ALK1)-dependent pathway [63]. That is, in addition to complexes containing two TGFBR2 and two TGFBR1 molecules, TGFB1 can form complexes with two TGFBR2, and one molecule each of TGFBR1 and ALK1. These latter receptor complexes can stimulate Smad1/5 phosphorylation and thereby inhibit TGFB1 actions mediated via TGFBR1 and Smad2/3 [63]. Whether or not LβT2 and/or gonadotropes express ALK1 has not been reported. However, it is possible that ALK1 expression in gonadotropes, but not LβT2 cells, may provide a mechanism for TGFB1 to antagonize endogenous activin B-dependent signaling (via Smad2/3) and hence decrease *Fshb *mRNA levels.

Although gonadotropes were purified to near homogeneity, it is possible that the inhibitory effects of TGFB1 were mediated indirectly through ligand action on contaminating cells in the cultures. For example, TGFB1 might stimulate FST synthesis by folliculostellate cells, which would then suppress the actions of endogenous activin B in gonadotropes [64]. However, if this were the mechanism of TGFB1 action, one might have anticipated greater inhibitory effects in the mixed rather than purified cultures where there are more folliculostellate cells, but the opposite was actually the case. That is, TGFB1 had greater suppressive activity in purified gonadotropes than in mixed cultures. Moreover, because activins stimulate FST production in primary pituitary cultures [65], one would predict that activin A would be less potent in stimulating *Fshb *mRNA levels in mixed than in purified cultures and this was in fact what we observed (Fig. 7). Collectively, these data suggest that the effects of TGFB1 on *Fshb *mRNA levels are likely not mediated via regulation of FST production, though we cannot rule out that possibility entirely.

The finding that TGFB1 inhibited *Fshb *expression in primary murine gonadotropes and mixed pituitary cultures is novel, and appears to contrast with data reported previously for the rat and sheep *Fshb *genes. For example, TGFB was shown to potently and dose-dependently stimulate FSH secretion from rat primary pituitary culture [66]. However, the TGFB preparation used in that study, which was purified from human platelets [67], did not function similarly to recombinant TGFB1 in similar assays [39,68]. Subsequent reports have failed to show major effects of TGFB1 on FSH in any dispersed pituitary culture. For example, TGFB1 did not affect ovine *Fshb *promoter-reporter activity in transgenic mice [51] or FSH secretion from rat primary pituitary cultures [39]. In addition, only minor stimulation was observed in primary ovine pituitary cultures [69].

Finally, it is also notable that we observed age-dependent increases in *Tgfbr2 *mRNA levels in purified gonadotropes. These data suggest that as mice mature, their gonadotropes may become more sensitive to the effects of TGFBs. The physiological significance, if any, of this change in receptor expression is not yet known, but is the subject of ongoing investigations. These data are nonetheless important in that they suggest that the low levels *Tgfbr2 *mRNA detected in gonadotropes from young mice are likely not due to contamination by other cell types. Instead, it appears that as gonadotropes age, the level of *Tgfbr2 *expression increases. Consistent with this notion is the lack of this receptor in LβT2 cells, which are thought to represent gonadotropes at an early stage of development. However, LβT2 cells were derived from a female mouse and the purified gonadotropes examined here were all from male mice, so it is possible that differences in *Tgfbr2 *mRNA levels may also reflect sex differences in receptor expression.

## Conclusion

The data reported here show that immortalized LβT2 cells lack the TGFB type II receptor, Tgfbr2, whereas the receptor appears to be expressed and functional in gonadotropes from male mice. As a result, TGFB1 (and likely all TGFB isoforms) is unable to regulate *Fshb *in LβT2 cells, but can inhibit transcription in primary murine gonadotropes. Because activins and TGFBs similarly activate Smads and TAK1, and both pathways contribute to activin A's stimulation of *Fshb *in rodents, it is surprising that TGFB would produce opposite effects to those of activins in purified gonadotropes. Nonetheless, as predicted, gonadotropes have evolved mechanisms for discriminating between the two classes of ligands. In the future, it will be critical to determine the mechanisms through which TGFBs inhibit *Fshb *in gonadotropes, particularly as animals age and *Tgfbr2 *expression increases. Importantly, the data presented here suggest that LβT2 cells may not provide the best model system in which to pursue this aspect of FSH regulation.

## Competing interests

The author(s) declare that they have no competing interests.

## Authors' contributions

AJG participated in the design of the study, performed all of the gonadotrope purification and real-time RT-PCR analyses, and drafted portions of the manuscript. DPP conducted RT-PCR and receptor expression analyses. WLM participated in the design of the study and critically revised the manuscript. DJB participated in the design of the study, performed many of the transfection and western blot experiments and analyses, and drafted significant portions of the manuscript. All authors read and approved the final manuscript.
